# Landscape of research, production, and regulation in venoms and antivenoms: a bibliometric analysis

**DOI:** 10.26633/RPSP.2021.55

**Published:** 2021-05-20

**Authors:** José Luis Di Fabio, María de los Ángeles Cortés Castillo, Elwyn Griffiths

**Affiliations:** 1 Independent consultant Washington D.C United States of America Independent consultant, Washington D.C., United States of America; 2 Independent consultant Mexico City Mexico https://orcid.org/0000-0003-0029-665x Independent consultant, Mexico City, Mexico, https://orcid.org/0000-0003-0029-665x; 3 Independent consultant in Vaccines and Biotherapeutics Kingston upon Thames United Kingdom https://orcid.org/0000-0002-5801-2448 Independent consultant in Vaccines and Biotherapeutics, Kingston upon Thames, United Kingdom, https://orcid.org/0000-0002-5801-2448

**Keywords:** Snakes, spiders, scorpions, venoms, antivenoms, bibliometrics, Serpientes, arañas, escorpiones, ponzoñas, antivenenos, bibliometría, Serpentes, aranhas, escorpiões, peçonhas, antivenenos, bibliometria

## Abstract

**Objectives.:**

To assess the productivity and visibility in research, clinical studies, treatment, use and production of antivenoms against poisonous snakes, scorpions and spiders.

**Methods.:**

Bibliometric analysis of research and other activities. Articles on venoms and antivenoms published between 2000 and 2020 were retrieved from the Scopus database. The records were analyzed by bibliometric indicators including number of documents per year, journals, authors, and citation frequency. VOSviewer^®^ v.1.6.13 was used to construct bibliometric networks for country co-authorships and co-occurrence of terms.

**Results.:**

Australia, Brazil, Costa Rica and India were among the six top countries with most documents and were selected for more detailed analysis. Costa Rica was the country with the largest percentage of its publications dedicated to antivenom production and venomics. Only a few papers dealt with the issues of quality, safety, and efficacy of antivenoms or the role of the national regulatory authorities. The use of VOSviewe*r*^®^ allowed visualization through joint publications of networking between countries. Visualization by co-occurrence of terms showed differences in the research carried out.

**Conclusions.:**

Working in a collaborative and coordinated manner these four countries could have a major impact on envenoming globally. Attention should be given not only to antivenom production but also to strengthening regulatory oversight of antivenom products.

## RESUMEN

**Objetivos.:**

Evaluar la productividad y la visibilidad en la investigación, los estudios clínicos, el tratamiento, el uso y la producción de antivenenos contra las picaduras de serpientes, arañas y escorpiones venenosos.

**Métodos.:**

Análisis bibliométrico de la investigación y de las otras actividades. Se tomaron los artículos sobre venenos y antivenenos publicados entre el 2000 y el 2020 en la base de datos de Scopus. Estos documentos se analizaron mediante indicadores bibliométricos como el número de documentos por año, revistas, autores o frecuencia en las citas. Se utilizó VOSviewer^®^ v.1.6.13 para crear una red bibliométrica para coautorías de países y coapariciones de términos.

**Resultados.:**

Australia, Brasil, Costa Rica e India estaban entre los seis primeros países con más documentos y se seleccionaron para un análisis más detallado. Costa Rica fue el país con el mayor porcentaje de sus publicaciones dedicadas a la producción de antivenenos y la venómica. Solo unos pocos artículos trataban los temas de la calidad, la seguridad y la eficacia de los antivenenos, o la función de las autoridades regulatorias nacionales. Gracias a VOSviewe*r*^®^ pudimos visualizar las publicaciones conjuntas de las colaboraciones entre países. La visualización por la coaparición de términos arrojó diferencias en la investigación realizada.

**Conclusiones.:**

Si estos cuatro países trabajasen de forma colaborativa y coordinada, podrían tener una repercusión mayor en los envenenamientos por picaduras en el mundo. El foco no debe ponerse solo en la producción de antivenenos, sino también en fortalecer la supervisión regulatoria de estos productos.

## RESUMO

**Objetivos.:**

Avaliar a produtividade e visibilidade em pesquisa, estudos clínicos, tratamento, uso e produção de antivenenos contra peçonhas de serpentes, escorpiões e aranhas.

**Métodos.:**

Análise bibliométrica de pesquisas e outras atividades. Artigos sobre venenos e antivenenos publicados entre 2000 e 2020 foram obtidos da base de dados Scopus. O conteúdo foi analisado segundo indicadores bibliométricos, como número de artigos por ano, periódicos, autores e frequência de citação. Utilizou-se o software VOSviewer^®^ v.1.6.13 para construir redes bibliométricas de coautoria de países e co-ocorrência de termos.

**Resultados.:**

Austrália, Brasil, Costa Rica e Índia figuraram entre os seis principais países com o maior número de artigos e, assim, foram selecionados para uma análise mais aprofundada. A Costa Rica teve a maior porcentagem de publicações dedicadas à produção de antivenenos e pesquisa em venômica. Apenas um pequeno número de artigos tratou de questões relacionadas à qualidade, segurança e eficácia dos antivenenos ou ao papel das autoridades nacionais reguladoras. O software VOSviewer^®^ permitiu visualizar, através das publicações conjuntas, as redes formadas entre diferentes países. A visualização por co-ocorrência de termos revelou diferenças nas pesquisas realizadas.

**Conclusões.:**

Trabalhando de forma colaborativa e coordenada, esses quatro países tiveram uma influência importante em nível mundial no campo de acidentes por animais peçonhentos. Deve-se atentar não apenas à produção de antivenenos, mas também ao fortalecimento da fiscalização regulatória destes.

Almost 2.5 million people annually undergo envenoming by snakes and other venomous animals such as spiders and scorpions of which nearly 120 000 die and 300 000 remain with some type of physical or psychological sequelae. This is such a public health problem that the World Health Organization (WHO) has included these medical emergencies within the neglected diseases for many tropical and subtropical countries (1). Although most of the cases occur in Africa and Asia, it is also a problem in Latin America and the Caribbean where between 80 000 to 130 000 cases and from 540 to 2 300 deaths occur annually (2,3). In 2011, it was reported that India had the highest number of deaths due to snake bites in the world with 35 000 to 50 000 people dying per year (4). It is important to mention that these figures are likely to be higher due to underreporting by countries (5). Most envenoming occur in rural populations and particularly affect women, children, and farmers in countries of low or middle income, where the health services and physicians are limited or nonexistent. Although snake envenoming is uncommon in Australia, the country has a long tradition of studying venoms, producing antivenoms, and assisting many countries in South East Asia (6).

The bites of venomous animals can cause acute medical emergencies that can range from severe paralysis with respiratory failure, bleeding, fatal hemorrhages, irreversible renal damage, and/or tissue necrosis with subsequent permanent disability that may lead to the amputation of limbs. These effects are caused mainly by toxins produced and secreted by the animal’s glands (7,8). Children suffer more severe effects than adults. Unlike other diseases, there is a highly effective treatment available, and most deaths and serious bite consequences of snakes, spiders, and scorpions can be prevented using specific antivenom sera.

In May 2018, the 71^st^ World Health Assembly adopted resolution WHA 71.5 formally providing the World Health Organization with a strong mandate to develop a comprehensive plan to support countries in implementing measures to increase access to effective treatment for people who get bitten by venomous animals (9). In 2019, WHO published the document *Snakebite Envenoming: A strategy for prevention and control* (10) aiming at reducing snakebite envenoming by 50% before the year 2030. Snake antivenom immunoglobulins had already been included in the WHO Model List of Essential Medicines since 2007 acknowledging their role in primary health care systems (11).

Australia, India and several countries in Latin America, like Brazil and Costa Rica, have developed an important infrastructure to study venoms and antivenoms of poisonous snakes, scorpions and spiders as well as to produce the corresponding antisera. This paper describes, through a bibliometric analysis, the productivity and visibility of the work that has been conducted in these selected countries for the last 20 years in research, clinical studies, treatment, use and production of antisera and how they could together make an important impact in tackling the global burden of envenoming.

## MATERIALS AND METHODS

**Source of information.** The bibliometric analysis was performed using documents published between January 2000 to March 2020 in journals indexed in Scopus (Elsevier BV Company, USA, https://www.scopus.com/), the largest abstract and citation database of scientific peer-review literature including more than 22 000 titles from international publishers. All types of documents were included in the analysis.

**Search strategy.** The literature search was conducted by the authors in Scopus for publications using the following terms in the title, abstract and keywords fields in the following sequence: (a) snake OR viper OR scorpion OR spider, (b) antiven* OR antiser*, (c) selection of period 2000 to 2020 and final selection of (d) Australia, (e) Brazil, (f) Costa Rica, and (g) India. The documents were exported to an Excel database where the validity of the search strategy was tested by manually reviewing retrieved articles, and for conducting further analysis. Each publication of the Excel database was assigned a country of authorship based on affiliation of authors (see Supplementary material).

**Data analysis.** Scopus provides tools to extract some bibliometric indicators that include number of documents per year, languages, countries, journals, authors, institutions, and citation frequency. It also allows the selection and analysis of the most cited articles.

For citation analysis, the top five publications per country, where the principal author is from the country in consideration, were selected. Multi-country publications (global) were not considered in the citation analysis.

Additional analyses of Scopus and Excel databases were conducted to identify publications related to antiserum/antivenom production, by searching for specific terms such as production, manufacture, and polyvalent sera. Another search was done in relation to the terms “omic” OR “proteomic” OR “venomic” in the titles and abstracts of the publications. The list of publications selected through Scopus was further searched for the following words: “quality” AND “safety” AND “efficacy” to select those publications that could have some link to regulatory issues.

Considering that “cross-reactivity” can be of great help for the development or improvement of antivenom manufacturing, the term was searched in the abstracts of all the publications from the four countries. In addition, the term “neutralization” was also searched, and the abstracts were revised manually to select those compatible with the concept of cross-reactivity.

**Visualization of bibliometric indicators**. For the construction and visualization of bibliometric networks the software VOSviewer^®^ v.1.6.13 for Microsoft Windows (Centre for Science and Technology Studies, Leiden University, The Netherlands; www.vosviewer.com) (12) was used. Out of the several types of networks that the software can produce, the following were selected for this study: (a) Network of co-authorship by countries were produced using a minimum of 5 publications; and (b) Network of co-occurrence of terms found in titles and abstracts, with a minimum of 15 occurrences in documents. The nodes and links in the visualizations were adjusted by weight by the number of publications and citations.

A VOSviewer^®^ thesaurus file for terms was created to merge terms that are synonyms, to correct spelling, to merge abbreviated terms with full terms, or to ignore terms that are not specific or are too general.

## RESULTS

**FIGURE 1.**
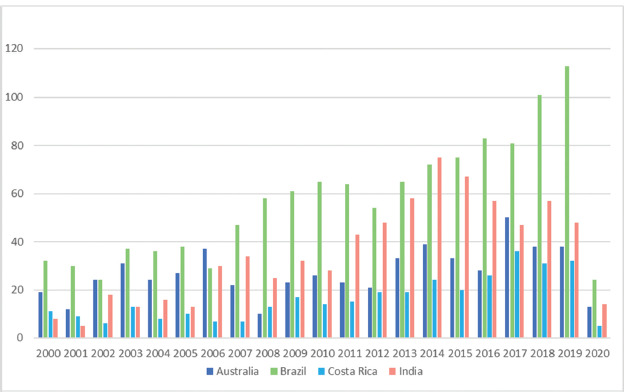
Publications by country and by year, 2000-2020

**Results from the search.** The first search, using the terms a) snake OR viper OR scorpion OR spider provided 101 736 documents, followed by b) antiven* OR antiser* that reduced the amount to 9 428 documents. By selecting the period 2000 to 2020 there was a further reduction to 6 375 documents. Australia, Brazil, Costa Rica and India were among the six top countries with most documents, together with the United States and the UK, and were selected for more detailed analysis. Figure 1 shows the number of publications published by these four countries yearly between 2000 and 2020.

After screening the information collected (e.g., removing duplicates, etc.), the following summary can be made: 560 articles from Australia were published in 153 journals, 1 183 articles from Brazil in 278 journals, 342 articles from Costa Rica in 83, and 719 from India in 286 journals. The five principal journals for each country analysis are shown in Table 1.

From the assignment of country of authorship based on affiliation of authors (see Supplementary material), an analysis was performed to identify multi-country publications. The principal five collaborators in multi-country publications for Australia were the United Kingdom, United States, Sri Lanka, Costa Rica and Papua New Guinea with 64, 56, 43, 22 and 18 publications, respectively. For Brazil, the collaborators were Costa Rica, United States, France, United Kingdom and Spain with 72, 61, 43, 42 and 29 publications, respectively. For Costa Rica, the five main collaborating countries were Spain, Brazil, United States, Colombia and the United Kingdom with 75, 72, 42, 38 and 37 publications, respectively. In the case of India, the collaboration was with United States, United Kingdom, Australia, Pakistan and Singapore with 48, 13, 11, 10 and 9 publications, respectively. Table 2 shows the five most cited publications from each selected country with and without self-citation.

**TABLE 1.** Main journals used for publication, by countries, 2000-2020

Journal of publication	Number	h-Index^1^	SJR (2018)^2^
**Australia**			
Toxicon	109	116	0.68
Toxins	34	56	1.03
Medical Journal of Australia	31	119	0.79
Plos Neglected Tropical Diseases	22	110	2.67
Clinical Toxicology	20	170	1.0
Percentage of total	**38.6%**		
**Brazil**			
Toxicon	347	116	0.68
Journal of Venomous Animals and Toxins including Tropical Diseases	60	22	0.72
Toxins	39	56	1.03
Revista da Sociedade Brasileira de Medicina Tropical	35	47	0.7
Journal Of Proteomics	30	92	1.15
Percentage of total	**43.2%**		
**Costa Rica**			
Toxicon	123	116	0.68
Journal of Proteomics	39	92	1.15
Toxins	20	56	1.03
Biologicals	12	51	0.56
Plos Neglected Tropical Diseases	11	110	2.67
Percentage of total	**59.9%**		
**India**			
Toxicon	43	116	0.68
Journal of Association of Physicians of India	35	53	0.20
Indian Pediatrics	21	46	0.34
Indian Journal of Critical Care Medicine	17	25	0.34
Wilderness and Environmental Medicine	15	37	0.47
Percentage of total	**18.2%**		

1 The h-index expresses the journal’s number of articles (*h*) that have received at least h citations; it quantifies both journal scientific productivity and scientific impact.

2 The SCImago Journal Rank (SJR) indicator is a measure of the scientific influence of journals that accounts for both the number of citations received by a journal and the importance or prestige of the journals where the citations come from.

**TABLE 2.** Five top cited publications per country, 2000-2020

Author	Title	Journal	Citations with self- citations	Citations excluding self- citations
**AUSTRALIA**				
Rash LD, Hodgson WC.	Pharmacology and biochemistry of spider venoms.	Toxicon. 2002;40(3):225-254	260	253
White J	Snake venoms and coagulopathy.	Toxicon. 2005;45(8):951-967	221	216
Tibballs J	Australian venomous jellyfish, envenomation syndromes, toxins and therapy	Toxicon. 2006;48(7):830-859	141	138
Vetter, I., Davis, J.L., Rash, L.D. *et al*	Venomics: a new paradigm for natural products-based drug discovery.	Amino Acids. 2011;40:15–28.	133	95
Fry BG, Wüster W, Ramjan SFR, Jackson T, Martelli P, Kini R.M	Analysis of *Colubroidea* snake venoms by liquid chromatography with mass spectrometry: evolutionary and toxinological implications	Rapid Commun Mass Spectrom. 2003;17:2047–2062	125	78
**BRAZIL**				
Zaher H, Grazziotin, FG, Cadle JE, Murphy RW, Moura-Leite JC, Bonatto SL	Molecular phylogeny of advanced snakes (Serpentes, Caenophidia) with an emphasis on South American Xenodontines: a revised classification and descriptions of new taxa.	Pap Avulsos Zool. (São Paulo).2009;49(11):115-153	244	221
Mors WB, Nascimento MC, Pereira BM, Pereira NA.	Plant natural products active against snake bite--the molecular approach.	Phytochemistry. 2000;55(6):627–642.	239	239
da Silva PH, da Silveira RB, Appel MH, et al	Brown spiders and loxoscelism	Toxicon. 2004;44(7):693-709	167	122
Teixeira CF, Landucci EC, Antunes E, Chacur M, Cury Y	Inflammatory effects of snake venom myotoxic phospholipases A2.	Toxicon. 2003;42(8):947-962	158	147
Soares AM, Ticli FK, Marcussi S, et al.	Medicinal plants with inhibitory properties against snake venoms.	Current Medicinal Chemistry. 2005;12(22):2625-2641	161	132
**COSTA RICA**				
Gutiérrez JM, Rucavado A.	Snake venom metalloproteinases: their role in the pathogenesis of local tissue damage	Biochimie. 2000;82(9-10):841-850	382	333
Gutiérrez JM, Ownby CL.	Skeletal muscle degeneration induced by venom phospholipases A2: insights into the mechanisms of local and systemic myotoxicity.	Toxicon. 2003;42(8):915-931	293	246
Gutiérrez JM, Rucavado A, Escalante T, Díaz C.	Hemorrhage induced by snake venom metalloproteinases: biochemical and biophysical mechanisms involved in microvessel damage	Toxicon. 2005;45(8):997-1011	284	224
Lomonte B, Angulo Y, Calderón L	An overview of lysine-49 phospholipase A2 myotoxins from crotalid snake venoms and their structural determinants of myotoxic action.	Toxicon. 2003;42(8):885-901	227	172
Calvete JJ, Sanz L, Angulo Y, Lomonte B, Gutiérrez JM	Venoms, venomics, antivenomics.	FEBS Lett. 2009;583(11):1736–1743	225	165
**INDIA**				
Mohapatra B, Warrell DA, Suraweera W, et al.	Snakebite mortality in India: a nationally representative mortality survey.	PLoS Negl Trop Dis.2011;5(4):e1018	250	220
Alirol E, Sharma SK, Bawaskar HS, Kuch U, Chappuis F.	Snake bite in South Asia: a review	PLoS Negl Trop Dis. 2010;4(1):e603	200	191
Alam MI, Gomes A.	Snake venom neutralization by Indian medicinal plants (*Vitex negundo* and *Emblica officinalis*) root extracts	Journal of Ethnopharmacology. 2003;86(1):75-80	144	140
Samy RP, Thwin MM, Gopalakrishnakone P, Ignacimuthu S.	Ethnobotanical survey of folk plants for the treatment of snakebites in Southern part of Tamilnadu, India	Journal of Ethnopharmacology. 2008;115(2):302-312.	132	131
Kemparaju K, Girish KS.	Snake venom hyaluronidase: a therapeutic target.	Cell Biochemistry and Function. 2006;24(1):7-12	113	100

**Antivenom production.** Results from the analysis of the publications of the four countries in relation to those that contained in their titles and abstracts terms related to antivenom production such as “antivenom production”, “manufacture”, “horse”, “camel”, “plasma”, etc., showed that Australia had 35 publications (6.3%), Brazil had 78 publications (6.6%), Costa Rica had 88 (25.7%) and India had 40 publications (5.6%).

**Publications related to omics, proteomics and venomics.** To identify the status of use and application of these technologies and concepts by the four countries, a search for the terms “omic” OR “venomic” OR “proteomic” was conducted in titles and abstracts of the publications. Costa Rica was the country with the largest percentage of its publications containing the terms, 21.9% in titles and 31.3% in abstracts. For Australia, Brazil, and India, these values were 5.4% and 12.9%, 4.5% and 10.7%, and 3.3% and 10.6%, respectively.

**Cross-reactivity.** Searching for studies on cross-reactivity, the keywords used were “cross-reactivity” and “neutralization”. The selected publications were analyzed manually and yielded 31 publications (5.5%) for Australia, 88 (7.4%) for Brazil, 63 (18.4%) for Costa Rica and 28 (3.9%) for India.

**Regulatory issues.** The search for publications covering regulatory issues identified 14 publications from Australia, 13 from Brazil, 33 from Costa Rica and 10 from India that once combined in one database, represented 61 publications. Looking into the contents of the abstracts, 23 publications were confirmed as being closer to the topic searched.

### Visualizations

**FIGURE 2.**
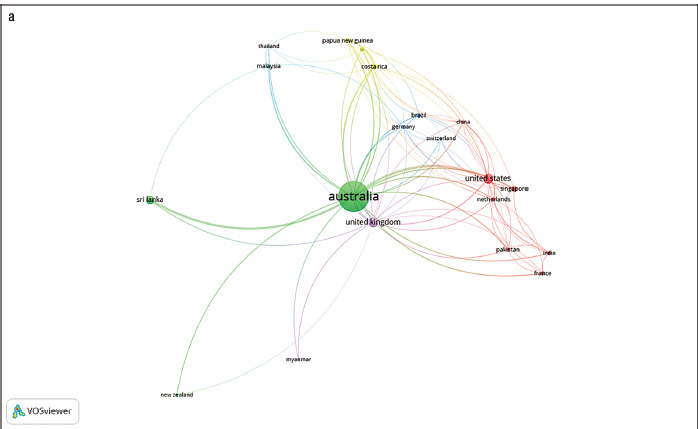

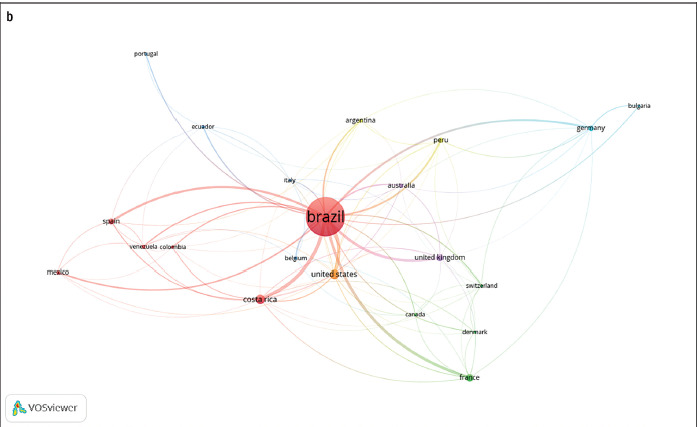

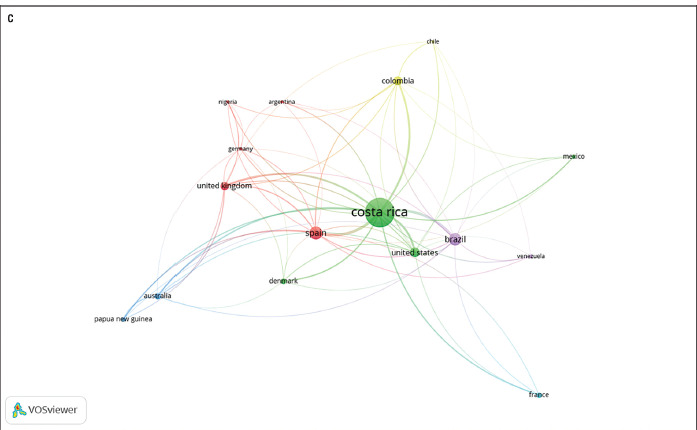

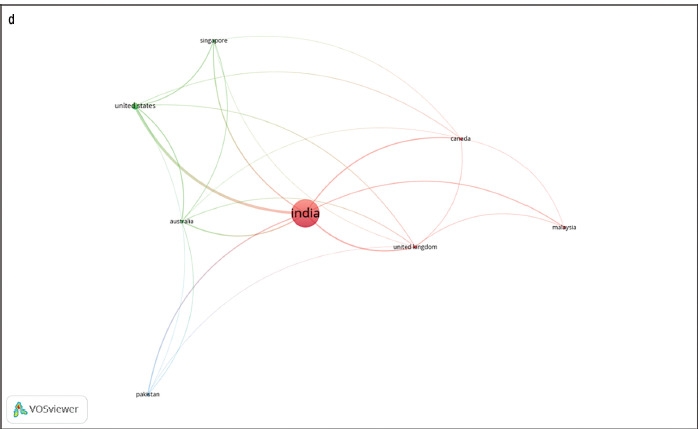
Visualization of country co-authorship maps (2a. Australia, 2b. Brazil, 2c. Costa Rica, 2d. India). The size of the node represents the number of publications of the country and the thickness of the lines indicate the strength of the linkage (co-authorship) with the other country

**Collaborative network among countries.** VOSviewer^®^ v.1.6.13 extracted countries from the Scopus databases for Australia, Brazil, Costa Rica, and India. Selecting for countries with at least 5 documents, the collaboration map based on co-authorship shown in Figure 2 was produced. The nodes represent the country, and the size of the node is related to the number of documents published. The links between the nodes indicate the collaboration (co-authorship), and the thickness of the lines the strength of the links (number of shared co-authorship).

**Co-occurrence of terms.** For the construction of the diagram of co-occurrence of terms, from each country individual database, the software identified initially in the titles and abstracts of the publications a large set of terms.

**FIGURE 3.**
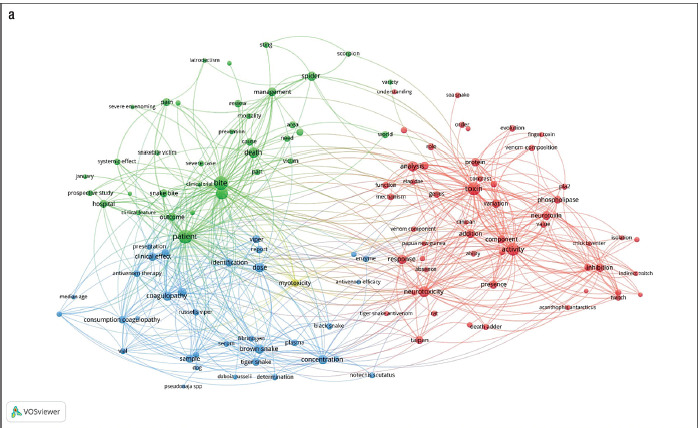

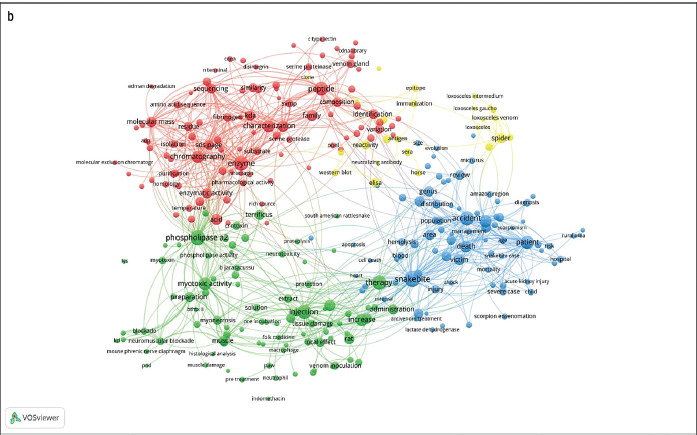

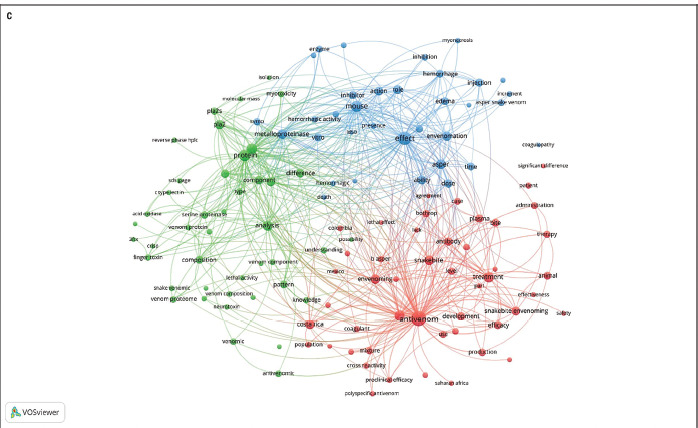

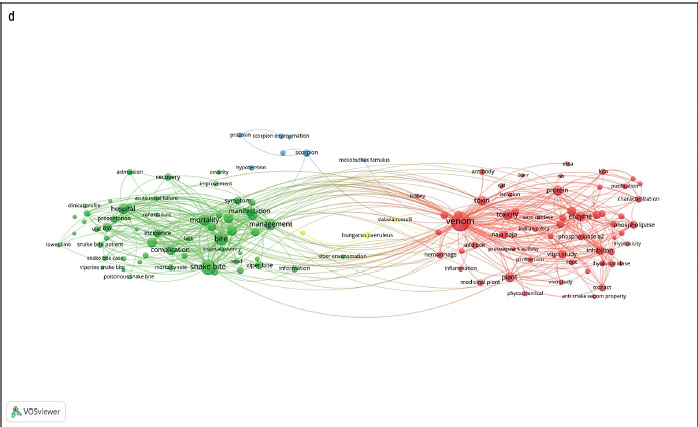
Visualization of co-occurrence of terms in titles and abstracts of country publications (3a. Australia, 3b. Brazil, 3c. Costa Rica, 3d. India). Each line represents at least 10 co-occurrences of both terms and in total, no more than 500 lines in each figure

In the case of Australia, from the initial total of 12 245 terms, 164 qualified as appearing in at least 15 publications (15 co-occurrences) by binary counting (presence or absence of the term is what counts). The number was further reduced to the 125 more relevant terms. In Figure 3a, three main clusters were generated by the software, where the red cluster contains terms related to venom, toxin, composition, and activity. The blue cluster contains terms related to clinical effects and antivenom therapy, while the green cluster to clinical data.

In the case of Brazil, from the initial total of 23 921 terms, 503 qualified as appearing in at least 15 publications (15 co-occurrences) by binary counting. The number was further reduced to the 300 more relevant terms. In Figure 3b, four clusters can be identified with the red cluster incorporating terms related to composition of venoms, physicochemical techniques used in the structural characterization, and activity. The blue cluster is related to clinical aspects of envenoming. The green cluster is more directed to the physiological activity and toxic activities of venoms and the smaller yellow cluster shows nodes related to spiders.

For Costa Rica, from the initial total of 8 523 terms, 144 appeared in at least 15 publications (15 co-occurrences) by binary counting, and the number was further reduced to the 125 more relevant terms. In Figure 3c, three clusters were identified with the red cluster showing terms related to antivenoms, production and use. The blue cluster incorporates terms related to the effect and activity of the venoms and the green cluster collects the terms related to venom analysis and composition, antivenomics, and venom proteomics.

Finally, for the case of India, from the initial total of 13 065 terms, 198 qualified as appearing in at least 15 publications (15 co-occurrences) by binary counting and the number was further reduced to the 125 more relevant terms. The pattern for India differs from those of the other countries and shows only two main clusters, the red cluster consolidating the terms related to the venom, composition, structure and activity and the green cluster identified with clinical symptoms and management (Figure 3d). A few isolated nodes (blue) are associated with scorpions.

## DISCUSSION

Bibliometric analysis is a tool that can be used to perform an objective evaluation of a scientific activity and to measure productivity (13,14), as well as to identify issues in need of attention. We linked this analysis to VOSviewer^®^ that provides us with the option of constructing and visualizing maps of networks of co-authorships and of the relevant terms extracted from titles and abstracts of the publications. With these tools we were able to follow the evolution and status of the field of envenoming in Australia, Brazil, Costa Rica, and India. Besides being among the top publishers of articles on snake, spider, and scorpion envenomation, these countries also have a tradition of antivenom production and use, and represent an important global resource for assisting other countries with similar problems but low research activity and access/availability to antivenoms.

It is important to mention that the present study only deals with envenoming by snakes, spiders and scorpions although it is recognized that other group of animals (15) produce venoms, and antivenoms and are being researched and produced. Since the year 2000, the selected countries have had an important and gradual increase of the number of publications. It is interesting to note that in more than 90% of the publications from Australia, Brazil and India, the principal author is from the country concerned, while for Costa Rica there is more interaction in authorship with authors from other countries and participation in close to 50% of publications from other countries. The journals *Toxicon, Toxins, Plos Neglected Tropical Diseases, Journal of Proteomics* and *Journal of Venomous Animals and Toxins including Tropical Diseases* were the main journals involved in publishing research on venoms and antivenoms.

Interestingly, two out of the five most cited papers for Brazil and India (see Table 2) were related to plant-based treatments which agrees with these two countries having the highest percentage of publications on this subject (India with 120 out of the 719 publications [16.6%] and Brazil with 105 out of the 1 183 publications [8.9%]). The number of publications from Australia and Costa Rica on this topic suggests that plant-based treatment is not an area of research interest in these countries.

The results of the analysis also show that the selected countries have made important advances in omics technologies such as proteomics, transcriptomics, and genomics (16–18), particularly Costa Rica, helping in the characterization of venom peptides and proteins, identifying the structures, chemical characteristics, genetics of venoms and antivenom cross reactions.

In general, there was significant work over the twenty-year period investigated on antivenom production, such as innovation and improvement of production processes, changing antigens used in immunizing animals (19) or using different animals for producing antisera (20–22). In the case of Costa Rica, 25% of the publications are related to this subject. These countries have also published important information on clinical characterization and treatment. Cross-reactivity is an element that has been studied by authors from the four countries, considering that lack of information on cross protection between products from different countries and regions is an obstacle that prevents the globalization of efforts in this area.

The visualization map of the most frequent terms found in the titles and abstracts of the publications of the four countries shows the main clusters of the research published (Figure 3a-d). Except for India, at least three defined clusters can be seen, one of them associated with structural characterization of the venoms, venomics and proteomics, another related to the toxicological and enzymatic activity of venoms, and a third one with clinical characteristics and treatment information. In the case of India, only two main separated clusters are seen corresponding to the clinical aspects and treatment and venom structure and characterization.

It was expected that using the three terms quality, safety, and efficacy together one would select publications linked to regulatory authorities or regulatory issues. While in several of these selected publications there is a concern that products are being used in many countries all over the world that lack the basic principles of quality and efficacy and there are serious doubts about their safety and efficacy (23), at the same time the role of a national regulatory authority with responsibility for the licensing and use of these antivenoms in these countries is hardly mentioned. There is one publication from the Global Snakebite Initiative where these roles are described together with WHO and a possible prequalification scheme for antivenoms (24). The quality assurance of these products, involving good manufacturing and clinical practices, and as well as other regulatory aspects associated with the production and use of these biologics is considered a key element, essential to assure their success in public health use. WHO has done extensive work to support countries by developing *Guidelines for the Production, Control and Regulation of Snake Antivenom Immunoglobulins* (25), which emphasize the pivotal role of national regulatory authorities in ensuring the quality, safety, and efficacy of antivenom products used in different countries.

The analysis also highlights the remarkable and intensive intercountry collaboration that has occurred in this field. This is seen mainly in the work of Australia, Brazil and Costa Rica which have developed extensive cooperation with several countries in assisting antivenom production and providing other technical assistance. This is clearly visualized in Figure 2. While Australia has concentrated efforts on countries in its subregion (Sri Lanka, Myanmar and Papua New Guinea) (26), Brazil has important collaborations in Mozambique (27) and in Latin America (28, 29) together with Costa Rica which has developed more worldwide collaborations as well as important interactions with Latin American countries. For example, Costa Rica has developed a polyvalent antivenom for Central America (30), a polyvalent antivenom for Sub-Saharan Africa (EchiTAB+ICP) (31,32) and assisted Papua New Guinea in antivenom development (33).

Recognizing further that snakebite envenoming has been categorized by WHO as a high priority neglected tropical disease, the WHA71.5 Resolution urged Member States to promote the transfer of knowledge and technology between Member States in order to improve the global availability of antivenoms and the effective management of cases and requested the WHO Director General to foster international efforts aimed at improving the availability, accessibility and affordability of safe and effective antivenoms for all (9).

One limitation of the analysis is that the articles considered were those that were published in journals indexed in Scopus. Although Scopus contains over 40 000 serial titles and all Med Line documents, it is possible that some national, local, and regional titles are not indexed and thus the results may not reflect the totality of research being carried out in the countries. Nevertheless, we believe most high impact publications were captured.

Results from this bibliometric analysis show that there is sufficient knowledge, expertise, and capacity to organize a coordinated international effort to address these issues. Australia has been helping countries in South East Asia; Brazil has supported African and Latin American countries; and Costa Rica has gone further to produce antivenoms for Central America, Sub Saharan Africa, and Papua New Guinea and has established an important transfer of technology with different countries. India has also its share of collaboration in its subregion. During the process to select the countries for the study it was clear that, measured by the number of publications, there are many other low- and middle-income countries where envenoming is a public health issue and the scientific and the clinical communities are not as involved in conducting relevant research. Promoting and establishing strategic alliances between these communities and involving other national or international stakeholders could be the basis for the development and application of knowledge into solving envenoming problems in particular contexts.

There is, however, a need for a centralized and concerted coordination of activities (34) with the assistance of WHO or the WHO Regional Offices such as the Pan American Health Organization, with funding from donors to improve and strengthen antivenom production capacities and regulatory oversight of antivenoms in selected countries, as well as to establish an appropriate supply and demand system with countries affected by envenoming. The pharmaceutical field has developed new technologies that can be easily implemented in the production of sera, improving yields, process control and effectiveness (35). In a gradual process that would allow maintenance of current production and avoid product shortages, the new initiatives should promote the fulfillment of requirements in good clinical, manufacturing, storage, and distribution practices to ensure the quality, safety, efficacy, and accessibility of these products, as well as their regulatory oversight. At the same time, efforts should be made to train countries in the rational use of antivenom and establish and/or strengthen surveillance, prevention, treatment, and rehabilitation programs as part of the public health system in the affected countries.

## Disclaimer.

The authors hold sole responsibility for the views expressed in the manuscript, which may not necessarily reflect the opinion or policy of the *RPSP/PAJPH* and/or PAHO.
